# The impact of Israel’s Front-of-Package labeling reform on consumers' behavior and intentions to change dietary habits

**DOI:** 10.1186/s13584-021-00482-w

**Published:** 2021-08-11

**Authors:** Shosh Shahrabani

**Affiliations:** Department of Economics and Management; Head of Research Authority, The Max Stern Yezreel Valley College, P.O. 1930600, Emek Yezreel, Israel

**Keywords:** Front-of-Package Labels, Health Belief Model, Nutrition

## Abstract

**Background:**

In January 2020, Israel launched a reform mandating Front-of-Package (FOP) labeling on food products. The current study examined the factors affecting consumers’ decision-making regarding the use of FOP labels a year after the reform was implemented.

**Methods:**

The survey was conducted between December 2020 and January 2021 and included a sample of 507 participants age 21 and over. The questionnaire included Health Belief Model (HBM) constructs related to food labeling, nutrition habits, media exposure and extent of support for the reform, frequency of using FOP labels, intention to change purchasing and consumption habits in the coming year, and personal details.

**Results:**

The study found that 58.5% reported using the FOP labels to some extent. In addition, 70% indicated willingness to change to healthier products in the coming year. The results of the analytical model confirm the validity of the HBM and the other behavioral constructs. In particular, the frequency of using FOP labels increases as the following factors increase: levels of perceived benefits and perceived importance of reading FOP labels, level of perceived importance of healthy nutrition, frequency of conforming to healthy nutrition, and support for the reform.

**Conclusions:**

The study's findings are important for understanding the impact of the new reform and for guiding future complementary actions to increase people’s motivation to use FOP labels. More advertisement about the FOP labels in the media and also through the HMOs is needed to increase people’s awareness of the reform. In addition, information about the reform provided to the public should emphasize the benefits and health implications of using FOP labels.

## Background

Over the last two decades, many countries have implemented food labeling schemes designed to encourage people to adopt healthier nutrition [1, 2]. Food labeling schemes are considered an intervention tool that may have positive long-term effects in encouraging the population to adopt healthy diets [3, 4]. This intervention, in turn, may reduce obesity and decrease the morbidity rates from chronic diseases such as diabetes and cardiovascular diseases [5].

Various studies have examined the effects of food labeling on consumer behaviors. A recent meta-analysis indicates that food labeling decreased consumer intake of energy by 6.6%, of total fat by 10.6% and of other unhealthy dietary choices by 13.0%, while increasing vegetable consumption by 13.5% [2]. In contrast, another review study indicates that Front-of-Package (hereafter FOP) labels may achieve only a small degree of success (< 2.0%) in persuading consumers to buy healthier foods [6]. Correa et al. [7] showed that in Chile, participants’ attention to and use of labels in their buying decision-making ranged from not paying any attention to the labels to relying on them as a quick shortcut. In addition, many mothers in Chile indicated they had changed their purchasing habits only when buying new products. In an experimental study in Austria, participants were shown pictures of products with different sugar levels. Some of these were marked with traffic light colors and some were not. The findings show that participants exhibited a higher intention to purchase products that were low in sugar when traffic light colors were used than when they were not [8]. Since the results of various studies are inconsistent and the effectiveness of food labeling remains unclear, more examination is needed to verify the effect of FOP labels on consumers' actual behavior.

In January 2020, Israel launched a reform mandating Front-of-Package (FOP) labeling on food products. The reform entails placing red warning labels on the front packaging of food products that contain values above the recommended threshold for saturated fat, sodium and sugar [9]. Additionally, some products are marked with a voluntary green label to indicate that their ingredients are on a list of healthier alternatives approved by the Ministry of Health. In fact, before the policy was enacted estimates indicated that 14.1% of food products from all food groups were eligible for negative FOP labels, 19.8% for positive FOP labels and 63.8% for no FOP labels at all [10]. The current study seeks to examine the impact of the new FOP labeling reform on consumers' decision-making regarding food purchases.

Obesity and unhealthy nutrition may lead to an increase in morbidity rates from chronic diseases such as diabetes. According to OECD data, more than half of Israel’s adult population is overweight or obese [11]. In addition, according to a survey by the Israel Center for Disease Control (ICDC), 81.9% of Israeli adults consume more than the recommended daily levels of sodium and 41.3% consume more than the recommended daily levels of saturated fats [12]. In addition, the average daily total sugar intake in Israel is 60 g for women and 71 g for men, higher than the recommendation for adults of 50 g of added sugar per day [12]. The 2020 FOP labeling reform is designed to motivate Israelis to adopt healthier nutrition patterns.

The current study, conducted a year after the new reform was launched in Israel, has two aims: The first aim is to map the main factors associated with whether Israelis use the information on FOP labels following the reform. The second aim is to examine factors that correlate with people’s intentions to use the new red/green FOP labels to change their shopping and dietary habits in the coming year. Understanding the factors that affect people’s use of FOP labels and their intentions to use the new labeling system has important implications for nutrition education and public health.

### Theoretical framework

The current study uses the Health Belief Model (HBM) [13] as its theoretical framework. The HBM model includes four constructs: *perceived benefits* of the preventive health action, *perceived barriers* of this action, *perceived susceptibility* to acquiring an illness or disease, and *perceived severity* as a reflection of an individual's beliefs regarding the seriousness of a health condition. The current study focused on the two constructs of perceived benefits and perceived barriers.

According to the HBM model, people are more motivated to engage in a particular health behavior when the perceived benefits of this behavior are higher (e.g., perceiving that FOP red/green labels provide useful information) and the perceived barriers for this behavior are lower (e.g., reading FOP labels takes very little time). The HBM model has been implemented in several studies examining decision-making regarding the use of nutrition labeling [e.g., 14–16]. These empirical studies show that the chances of using the information on nutrition labels are positively correlated with the perceived benefits [14, 15] and negatively correlated with the perceived barriers to using the information on the labels [14].

Other psychological factors that correlate with the preventive health behavior of using food labels include the perceived importance of using food labels and the perceived importance of healthy nutrition (e.g., "It is important to take nutrition into account in food shopping"). Higher levels of perceived importance of using food labels and of healthy nutrition are associated with more frequent use of food labels [14, 16].

The current study uses the theoretical HBM framework principles and health behavior constructs to examine Israelis’ frequency of using FOP labels and their intentions to use the information provided by the red/green food labeling system in the upcoming year.

### Hypotheses

Based on the literature review, the research hypotheses are:

#### H1

The frequency with which people use the FOP labels on food products and the level of their intentions to use them in the coming year to change their shopping and dietary habits will increase under the following conditions: (a) The *perceived benefits* of using FOP labels are higher; (b) The *perceived barriers* to using FOP labels are lower; (c) The perceived importance of reading FOP labels is higher; (d) The perceived *importance of nutrition* and choosing a healthy diet increases; (e) The level of positive attitudes toward the FOP labeling reform increases.

## Methods

The Institutional Review Board of the Max Stern Yezreel Valley College approved the current research. All participants gave their verbal informed consent, which was witnessed and formally recorded (see Participants' Consent in “Appendix 1”).

### Sample and sampling

Between December 12, 2020 and January 21, 2021, the B.I. and Lucille Cohen Institute for Public Opinion Research, a professional polling company, surveyed a representative sample of the adult population in Israel age 21 and over from the Jewish and non-Jewish sectors. The representativeness of the sample was examined based on distribution of the following socio-demographic characteristics relative to their distribution in the general population according to Central Bureau of Statistics (CBS) data: age, gender, religion, level of religious observance, and level of education. Constructing the sample included two stages: first, building a random sample of households according to the age criteria, and second, locating telephone numbers for the sampled households. To ensure sample representativeness and high response rates, the polling company conducted the telephone survey five days a week, at different times and on different days. Repeated trials were conducted on specific dates determined in accordance with the interviewees. In addition, the names of respondents who were not reached were recorded on a special list to control repeated trials (up to five repetitions per household) on different days and hours. The firm also made attempts to persuade those who initially refused to participate in the survey. The gross sample included 998 households that constituted the research population. The final sample included 507 respondents (50.8% response rate) who were interviewed by interviewers. Of the gross sample, 69 (0.7%) did not answer the phone, 411 (41.2%) refused to participate, and 11 (1.1%) answered partially and were eliminated from the final sample.

The sample (participants age 21 and above) included 51.7% women and 48.3% men; 80.9% were Jews and 19.1% were Arabs and others; 47.0% were between the ages of 21 and 44, 31.8% were between the ages of 45 and 64, and 21.1% were age 65 and over; 36.9% had up to 12 years of education and 63.1% had more than 12 years of education. In comparison, according to Central Bureau of Statistics (CBS) data, the adult Israeli population over the age of 20 consists of 51.3% women and 48.7% men; 77.1% are Jews and 22.9% Arabs; 52.5% are between the ages of 20 and 44, 29.5% are between 45 and 64, and 17.9% are age 65 and above; 40.3% have up to 12 years of education and 59.7% have more than 12 years of education [17].

### Questionnaire

The questionnaire included the following parts:

1. Socio-demographic information: Gender; age; education; marital status; nationality; degree of religious observance (1 = not at all religious, 5 = very religious); year of immigration; household income (1 = above average, 5 = much lower than average); place of residence; and number of persons in household.
2. HBM constructs related to food labels, based on [14,18] (see Table 5 “Appendix 2”). The scale ranged from 1 = strongly agree to 5 = strongly disagree. The constructs are: perceived benefits (e.g., “FOP red/green labels provide useful information”); perceived barriers (e.g., “reading FOP red/green labels takes more time than one can spare”); perceived importance of reading FOP red/green labels (e.g., "using red/green labels to choose food products is better than relying on one’s own knowledge about what is in them"); perceived importance of nutrition (e.g., "It is important to take nutrition into account when shopping for food"); and health motivation.
3. Health and nutrition habits: Perceived health status (1 = very good, 5 = very bad); chronic illness (Yes/No); extent to which participant maintains a healthy diet with low sugar/salt/saturated fat (0 = not at all, 6 = to a large extent); extent to which participant maintains a normal body weight relative to age and height (1 = not at all, to 5 = to a very large extent);
4. Media exposure to the reform: Has participant heard about the new red/green FOP labels in Israel (yes/no); extent of exposure to media messages regarding importance of consuming heathy food and avoiding products with red labels (1 = not at all, 5 = to a large extent); extent of positive attitude toward the reform (extent of support for this reform and extent of perceived importance of the reform) (1 = not at all, 5 = to a large extent); percentage increase in price participant is willing to pay to purchase an alternative product that does not have a red label (not at all/up to 5%/up to 10%/up to 15%/more than 15%).
5. Frequency of using FOP red/green labels on purchased and consumed food products was measured on a scale of 1–5 (1 = not at all, 5 = always) as a complex variable that included four items: (a) “I look for the red and green labels on food products"; (b) "I avoid buying products that have a red label and try to buy alternative products"; (c) "I avoid buying products with two or more red labels"; (d) "I try to buy food products that have a green label or products without a label." Degree of intention to change buying and consumption habits in the next twelve months to healthier products in the wake of the reform was measured on a scale of 1–5 (1 = not at all, 5 = to a very large extent).

**Table 1:** Survey data—Frequency of using FOP labels and intention to change food buying habits following the reform according to socio-demographic and other characteristics

	Entire sample (N = 487)^a^	Using FOP labels^b^			Intention to change habits in the next year to healthier products following the reform^c^		
	%	Not at all/ to a small extent (N = 187) (%)	Medium extent (N = 161) (%)	Very large/ large extent (N = 139) (%)	Not at all/ to a small extent (N = 140) (%)	Medium extent (N = 114)(%)	Very large/ large extent (N = 203) (%)
*Gender*							
Women	51.9	36.6	32.3	31.1	27.0	25.8	47.2
Men	48.1	40.3	33.9	25.8	34.4	24.1	41.5
*Age*							
21–39	35.4	39.9	35.8	24.3	30.2	27.2	42.6
40–59	35.6	39.1	31.0	29.9	28.7	25.1	46.1
> 60	29.0	35.7	32.1	32.1	33.6	21.9	44.5
*Religion*							
Jewish	82.8	39.5	32.8	27.8	33.1	24.8	42.1*
Arab	17.2	33.3	34.5	32.1	18.9	28.4	52.7
*Marital status*							
Married	70.7	38.7	32.3	29.1	28.0	26.5	45.5
Not married	29.3	37.8	35.0	27.3	36.6	21.4	42.0
*Education*							
Up to 12 years	36.9	34.8	29.2	36.0*	27.2	23.7	49.1
More than 12 years	63.1	40.3	35.4	24.4	32.4	25.8	41.8
*Income*							
Below average	42.4	35.3	37.5	27.2	29.6	27.8	42.6
Average	27.0	41.0	24.8	34.2	26.6	23.9	49.5
Above average	30.5	40.5	35.9	23.7	33.1	24.4	42.5
*Residential region*							
Tel Aviv and center	43.0	41.1	30.9	28.0	29.4	26.3	44.3
Other	57.0	36.4	34.9	28.7	32.0	23.6	44.4
*Extent of religious observance*							
Ultra-orthodox	9.1	30.2	34.9	34.9	22.0	34.1	43.9
Not ultra-orthodox	90.9	39.2	32.8	28.0	31.4	24.3	44.4
*Number of persons in household*							
2 or below	38.6	37.1	34.9	28.0	34.9	24.3	40.8
3 and above	61.4	39.3	31.9	28.9	28.1	25.3	46.7
*Maintain a normal body weight*							
Not at all/small/medium extent	36.7	48.6	32.8	18.6***	41.7	26.8	31.5**
Large/very large extent	63.3	32.7	33.3	34.0	23.9	23.9	52.3
*Chronic illness*							
No	75.4	38.1	34.8	27.1	30.8	23.6	45.5
Yes	24.6	40.2	28.2	31.6	30.2	28.3	41.5

*p < 0.05; **p < 0.01; ***p < 0.001

^a^Number of participants (N = 487) does not include those who answered “do not know” to one of the two dependent variables: using FOP labels and intention to change habits in the coming year

^b^Using FOP labels variable is the average of three items. Each item was measured on a 5-point scale categorized as: Not at all/to a small extent: 1–2.4; Medium extent: 2.5–3.5; Very large/large extent: 3.6–5

^c^Number of participants (N = 457) does not include those who reported they do not intend to change habits since they already maintain a healthy diet

The questionnaire was translated into Hebrew by the author and then back-translated by an English editor. In addition, a professional company translated the Hebrew version into Arabic and back into Hebrew. In the first stage, a pilot questionnaire was administered to 50 individuals, and after improvements were made, the final format was developed.

### Statistical data analysis methods

Data were analyzed with SPSS version 27. Chi-square analyses were used to assess the associations between the socio-demographic, attitudinal and HBM variables, and the two dependent variables: using FOP labels and intention to change shopping habits in the next twelve months to healthier products in the wake of the reform. Internal consistency of the study variables was calculated with Cronbach’s alpha. The study variables were described by means and standard deviations, and Pearson intercorrelations were calculated between them.

A logistic regression model was calculated for the intention to change shopping habits in the coming year, while a multiple linear regression model was calculated for the dependent variable of using FOP labels. Significance level was set at *p* = 0.05, and Bonferroni correction for multiple comparisons was used when needed.

## Results

The results show that 49.2% of the sample reported hearing about the food labeling reform in the media during the last year to a medium or large extent, 24.0% heard about the reform to a small extent, while more than a quarter of the sample (26.8%) reported they had not heard about it in the media at all. More specifically, the rates of those who were not exposed to the reform in the media were significantly higher among ultra-Orthodox people than among those who are not ultra-Orthodox (43.2% vs. 24.8%); among those with less than 12 years of education than among those with higher education (35.6% vs. 21.5%); and among those with lower than average incomes (35.3%) than among those with average (15.4%) or above average incomes (19.8%).

Most of the participants (90.5%) supported the food labeling reform to a medium/large or very large extent and most of them agreed to a medium/large or very large extent that the reform is important (92.6%).

The findings indicate that 35.9% of the sample reported that they often or always use the red/green FOP labels and they avoid buying products with red labels, 22.6% reported that they sometimes use the FOP labels, while 41.5% stated they rarely or never use the FOP labels. The findings also indicate that the majority of the sample (51.1%) reported changing their buying habits to healthier products in the wake of the reform (to a medium/large/very large extent). Yet, 47.8% reported they had almost not changed their purchasing habits or had not changed them at all. Nevertheless, 9% of this group reported they had not changed their habits since they already were in the habit of purchasing healthy food. The percentage of those who reported changing their buying habits to healthier products was higher among the following groups: (a) those with up to 12 years of education compared to those with more than twelve years of education (59.1% versus 54.7%, respectively), and (b) those in the 40–59 age group (60.6%) compared to those between the ages of 21 and 39 (50.7%) and those in the 60 and above age group (57.7%).

The study also found that 69.3% said they are willing to change their food purchasing and consumption habits to healthier products in the coming year, while 30.6% said they do not intend to change their habits or probably will not. In addition, 63.5% of the sample indicated they are willing to pay extra for alternative products that are not marked by a red label.

As for nutrition habits, most of the participants reported maintaining a balanced and healthy diet (65.6%). However, more than 20% of the sample reported often or always consuming products that are high in salt (20.7%), sugar (31.3%), and saturated fats (31.7%). Those who reported conforming to a healthy diet very often or always included more women than men (67.3% versus 63.8%, respectively) and more people age 60 and above (75.4%) than people age 40–59 (63.2%) and age 21–39 (60.1%).

Table 1 summarizes the distribution of the entire sample according to different socio-demographic and other personal characteristics (column 3). Additionally, Table 1 compares the percentage of each characteristic according to: (a) frequency of using FOP red/green food product labels, a complex variable with Cronbach’s alpha of 0.91, and (b) degree of intention to change buying and consumption habits in the next twelve months to healthier products in the wake of the reform. The number of participants who intend to change their buying habits (N = 457) does not include the 26 who reported they do not intend to change their habits since they already maintain a healthy diet.

The findings shown in Table 1 indicate that the frequency of using FOP red/green labels on purchased and consumed food products is significantly higher among those with up to 12 years of education compared to those with more than 12 years (36.0% versus 24.4%, *p* < 0.05) and among those who usually maintain a normal body weight compared to those who rarely or never maintain a normal weight (34.0% versus 18.6% *p* < 0.001). In addition, the findings indicate that the degree of intention to change buying and consumption habits in the next twelve months to healthier products following the reform is higher among Arabs than among Jews (52.7% versus 42.1% *p* < 0.05) and among those who usually maintain a normal body weight than among those who rarely or never maintain a normal weight (52.3% versus 31.5% *p* < 0.01).

### Results for attitudes and HBM categories

Table 2 shows the distribution of the sample according to the attitude variables and the HBM categories. The Cronbach's alpha coefficients are reported in Table 5 in the “Appendix 2”. The table also shows the percentage of each variable according to the frequency of using FOP labels on food products and the degree of intention to change shopping habits in the next twelve months. The extent to which FOP labels affect buying decisions was classified into three categories: never or rarely (1–2.4), sometimes (2.5–3.5), often or always (3.6–5). The intention to change shopping habits was classified into three categories: not at all or to a small extent, to a medium extent, and to a large or very large extent. The associations between the dependent variables and the study variables were examined using Chi-square analyses.

**Table 2:** Distribution of HBM variables, attitudes and other variables across Categories of FOP label use and intention to change shopping habits after the reform

	Entire sample (N = 487)^a^	Using FOP labels^b^			Intention to change shopping habits^c^		
	%	Not at all/ to a small extent (N = 187)	Medium extent (N = 161)	Very large/large extent (N = 139)	Not at all/to a small extent (N = 140)	Medium extent (N = 114)	Very large/large extent (N = 203)
*Benefits*							
Not agree at all/agree to small/medium extent	49.7	51.3	29.7	19.1***	41.4	24.3	34.2***
Agree to a large/very large extent	50.3	24.1	36.9	39.0	19.7	25.8	54.6
*Barriers*							
Not agree at all/agree to small extent	72.1	36.1	31.9	31.9	31.2	23.2	45.5
Agree to medium/large/very large extent	27.9	34.9	40.3	24.8	23.1	31.4	45.5
*Perceived importance of reading FOP red/green labels Perceived importance of reading FOP red/green labels*							
Not agree at all/agree to small extent	45.5	48.1	28.3	23.6***	38.1	22.7	39.2**
Agree to a medium /large/very large extent	54.5	27.7	37.9	34.4	23.3	27.3	49.4
*Importance of healthy nutrition*							
Not agree at all/agree to small/medium extent	23.1	59.3	34.5	6.2***	47.7	28.0	24.3***
Agree to large/very large extent	76.9	32.1	32.6	35.3	25.4	24.0	50.6
*Positive attitudes*							
Not agree at all/agree to small/medium extent	27.7	69.4	24.6	6.0***	50.4	29.3	20.3***
Agree to large/very large extent	72.3	25.9	36.5	37.6	23.6	23.3	53.0

^*^p < 0.05; **p < 0.01, ***p < 0.001

^a^Number of participants (N = 487) does not include those who answered “do not know” to one of the two dependent variables: using FOP labels and intention to change habits in the coming year.

^b^Using FOP labels variable is the average of three items. Each item was measured on a 5-point scale categorized as: Not at all/to a small extent: 1–2.4, Medium extent: 2.5–3.5; Very large/large extent: 3.6–5

^c^Number of participants (N = 457) does not include 26 who reported they do not intend to change habits since they already maintain a healthy diet and 24 participants who answered "do not know"

The results in Table 2 indicate that the following HBM categories were significantly and positively associated with the extent to which FOP labels affect buying decisions and with intentions to change shopping habits in the next year: perceived benefits, perceived importance of reading FOP labels, perceived importance of healthy nutrition, and positive attitudes toward the reform. In other words, higher levels of perceived benefits, perceived importance of reading FOP labels, perceived importance of healthy nutrition, and positive attitudes toward the reform are associated with more frequent use of FOP labels and higher intentions to change buying habits in next year following the reform.

### Analytical model results

Table 3 summarizes the results of the multiple linear regression model for the dependent variable of frequency of using FOP labels, while Table 4 summarizes the results of the logistic regression model for the intention to change habits of shopping for food products in the coming year.

**Table 3:** Multiple hierarchical linear regression model for using FOP labels (*N* = 487)

	B	SeB	β	*p* value
*Step 1*				
Gender (male)	0.13	0.10	0.06	0.162
Age	0.01	0.00	0.01	0.804
Ethnicity (Jews)	− 0.24	0.12	− 0.07	0.057
Education level (academic)	− 0.19	0.10	− 0.08	0.052
Health status	0.04	0.11	0.01	0.720
*Step 2*				
Body weight	0.13	0.10	0.05	0.187
Health motivation	0.07	0.05	0.06	0.157
Importance of healthy nutrition	0.20	0.08	0.11	0.012
Healthy nutrition	0.40	0.05	0.32	< 0.001
*Step 3*				
Media exposure	0.10	0.04	0.09	0.026
Positive attitudes	0.30	0.06	0.24	< 0.001
*Step 4—HBM*				
Perceived benefits	0.18	0.06	0.14	0.002
Perceived barriers	0.01	0.09	0.01	0.955
Perceived importance of reading FOP labels	0.23	0.10	0.09	0.024

Step 1: Adj. *R*^2^ = .029, *p* = 0.003. Step 2: ΔAdj. *R*^2^ = .231, *p* < 0.001. Step 3: ΔAdj. *R*^2^ = .101, *p* < 0.001. Step 4: ΔAdj. *R*^2^ = .026, *p* < 0.001. Total model: Adj. *R*^2^ = .387, *F*(14, 472) = 20.58, *p* < 0.001

**Table 4:** Logistic regression model for intention to change habits of shopping for food products in the year following the reform (*N* = 457)

	B	SeB	OR (95%CI)	*p* value
*Step 1*				
Gender (male)	− 0.09	0.25	0.91 (0.56, 1.49)	0.711
Age	0.01	0.01	1.00 (0.98, 1.02)	0.847
Ethnicity (Jewish)	− 0.73	0.35	0.48 (0.24, 0.96)	0.038
Education level (academic)	− 0.36	0.26	0.70 (0.42, 1.17)	0.174
Health status	0.26	0.27	1.30 (0.76, 2.23)	0.341
*Step 2*				
Body weight	0.40	0.25	1.49 (0.91, 2.45)	0.114
Health motivation	0.27	0.13	1.32 (1.02, 1.69)	0.032
Importance of healthy nutrition	0.21	0.19	1.23 (0.85, 1.78)	0.279
Healthy nutrition	0.18	0.14	1.20 (0.92, 1.57)	0.188
*Step 3*				
Media exposure	0.09	0.12	1.09 (0.87, 1.37)	0.455
Positive attitudes	0.30	0.14	1.36 (1.03, 1.78)	0.029
*Step 4—HBM*				
Perceived benefits	0.42	0.16	1.52 (1.12, 2.06)	0.007
Barriers	0.21	0.25	1.23 (0.76, 2.00)	0.400
Perceived importance of reading FOP labels	0.34	0.25	1.41 (0.86, 2.32)	0.177

Step 1: χ^2^(5) = 12.25, *p* = 0.031, Nagelkerke’s *R*^2^ = 0.042. Step 2: χ^2^(4) = 31.61, *p* < 0.001, Nagelkerke’s *R*^2^ = .102. Step 3: χ^2^(2) = 14.73, *p* < 0.001, Nagelkerke’s *R*^2^ = .046. Step 4: χ^2^(3) = 14.42, *p* = 0.002, Nagelkerke’s *R*^2^ = .042. Total model: χ^2^(14) = 73.01, *p* < 0.001, Nagelkerke’s *R*^2^ = .232

Each model was calculated hierarchically and included four steps. The first step included the demographic variables of gender (0—female, 1—male,), nationality (0—Arabs, 1—Jews), education level (0—up to 12 years, 1—over 12 years), and health status (0—good and lower, 1—very good/excellent). The second step included variables related to health and nutrition: attempting to maintain proper body weight (0—moderate extent or less, 1—large extent); health motivation (5-point scale ranging from 1—not at all to 5—to a large extent); attribution of importance to healthy nutrition (5-point scale ranging from 1—not at all to 5—to a large extent), and extent of maintaining healthy nutrition (7-point scale ranging from 0—never to 6—always). The third step included variables related to the FOP labeling reform: extent of media exposure to the reform (5-point scale ranging from 1—not at all to 5—to a large extent), and positive attitudes toward the reform (5-point scale ranging from 1—not at all to 5—to a large extent). The fourth and final step included the HBM variables of perceived benefits (5-point scale ranging from 1—not at all to 5—to a large extent), barriers (0—no, 1—yes), and perceived importance of reading FOP labels (0—no, 1—yes).

The step-wise regression analysis that included four steps was necessary to show the added explained variance of the variables included in each step. In particular, the HBM variables were entered last to examine whether they made a significant contribution even after basic personal variables and variables of the FOP labels reform were accounted for.

The results in Table 3 show that the following variables were significantly associated with frequency of using FOP labels: attribution of importance to healthy nutrition; adherence to healthy nutrition; media exposure to the reform; positive attitudes toward the FOP labeling reform; and the HBM variables of perceived benefits and perceived importance of reading FOP labels. More specifically, the chances of using the FOP labels on food products increase as the level of perceived importance of healthy nutrition increases, the frequency of adhering to healthy nutrition increases, the extent of media exposure to the reform increases, the level of positive attitudes toward the FOP labeling reform increases, and the levels of perceived benefits and perceived importance of reading FOP labels increase.

The results in Table 4 show that the following variables were significantly associated with the intention to change habits in shopping for food products in the coming year: nationality, health motivation, positive attitudes toward the FOP labeling reform, and the HBM variable of perceived benefits. More specifically, the intention to change habits in shopping for food products in the coming year was higher among non-Jews than among Jews, among those with higher levels of health motivation, among those with higher levels of positive attitudes toward the FOP labeling reform and among those with a higher level of perceived benefits from the FOP labeling reform.

It is important to note that the HBM variables made a significant contribution to actual changes in shopping habits (Nagelkerke’s *R*^2^ = 0.041, *p* = 0.001), intentions to change shopping habits (Nagelkerke’s *R*^2^ = 0.042, *p* = 0.002), and reading FOP labels (ΔAdj. *R*^2^ = 0.026, *p* < 0.001), beyond the contribution of all variables entered in the preceding steps.

## Discussion

The current study examined the factors affecting people’s decisions to make use of the FOP labeling reform in Israel a year after its implementation. The results show that most of the participants (73%) reported having heard about the new reform in the media, while more than a quarter (27%) reported not hearing about it in the media at all. The analysis shows that the rates of those who were not exposed to the reform in the media were significantly higher among the ultra-Orthodox population, among those with less than 12 years of education and among those with a lower than average income.

One interesting result is that more than 90% of the participants supported the reform and perceived it as important to a medium/large or very large extent, similar to the findings of a study conducted in Israel before the reform was instituted [19]. Nevertheless, the results show that only 58.5% reported using the FOP labels to some extent, while 41.5% reported rarely or never using the FOP labels. Moreover, 47.8% of the participants reported they had practically not changed their purchasing habits or had not changed them at all. Before the reform was implemented, more than 20% reported often or always consuming products that are high in sugar, saturated fats, and salt [15]. Yet in the present study, the results show similar rates despite consumers' yearlong exposure to the labels. Nevertheless, about 70% of the sample said they would be willing to change their food purchasing and consumption habits to healthier products in the coming year. All these findings may imply that the policy has had little impact on actual behaviors, at least among part of the population, despite self-reported interest or intentions to change habits.

According to a Myers–JDC–Brookdale study conducted in Israel in 2019 before the reform was implemented, Arab households purchased a greater percentage of some products, such as sweet beverages, sweetened dairy items and snacks, than Jewish households, and ultra-Orthodox Jewish households purchased a greater percentage of these products than Jewish households that were not ultra-Orthodox [19]. The results of the current study conducted a year after the reform was implemented did not reveal any significant differences between population groups in frequency of using FOP labels. In addition, in 2019 intentions to change consumption habits following the reform were found to be higher among ultra-Orthodox, Arab and young families [19]. The results of the current study in 2021 show that intentions to change habits in the next year to healthier products following the reform were significantly higher among Arabs than among Jews. Yet the actual self-reported frequency of using FOP labels among Arabs was much lower than their intentions to use FOP labels and did not differ significantly from the frequency reported by Jews. A possible explanation is that the food consumption behavior of various population groups has changed not only because of the FOP labeling reform but also due to the simultaneous occurrence of the COVID-19 pandemic in Israel.

The findings of the bivariate analysis (Chi-square analysis) indicate that the frequency of using the FOP red/green labels on purchased and consumed food products is significantly higher among those with lower education levels and among those who usually maintain a normal body weight. Yet, the multivariate analysis (regression analyses) did not show any significant differences in the frequency of using the FOP labels between those with high and low education levels. This result with respect to education levels is in line with a study from Chile, which found that the decrease in purchases of high-in-sugar beverages after the 2016 mandated FOP warning labels was similar among households with high and low education levels [20]. A possible explanation for this lack of significant differences in frequency of using the FOP labels according to education levels may be that the FOP labels are quite easy to read and understand and do not need the high levels of health literacy required to understand the nutrition labels on food products. Indeed, those with higher levels of education have higher health literacy and use nutrition labels more frequently than those with lower levels of education [15].

The results of the analytical model (regression analyses) confirm the validity of the HBM and other behavioral constructs. In particular, the findings indicate that the frequency of using FOP labels on food products increases as the following factors increase: level of perceived importance of healthy nutrition, frequency of consumption of healthy nutrition, extent of media exposure to the reform, level of positive attitudes toward the FOP labeling reform, and levels of perceived benefits and perceived importance of reading FOP labels. These results are in line with hypotheses H1(a), (c), (d) and (e). They are also compatible with the findings of previous studies showing that higher levels of perceived importance of reading food labels and perceived importance of healthy nutrition are associated with higher frequency of using nutrition food labels in the US and in Thailand [14, 16].

In addition, the intention to change habits in shopping for food products in the coming year was higher among non-Jews than among Jews, in line with the findings that frequency of using nutrition information on food product labels before the reform was higher among non-Jews than among Jews [15]. Moreover, our findings show that the intention to change habits in shopping for food products was higher for those with higher levels of health motivation, those with higher levels of positive attitudes toward the FOP labels reform and those with a higher level of perceived benefits from the FOP labeling reform.

The results of the current study suggest that complementary policy steps are needed to increase people’s motivation to use FOP labels after the reform. First, since more than a quarter (27%) reported they had not heard about the reform in the media at all, more advertisement about the FOP labels is needed, specifically via channels targeting ultra-Orthodox and low income population groups (e.g., through HMOs and Tipat Halav family health centers), in order to increase people’s awareness of the reform. Second, information about the reform provided to the public should emphasize the benefits and health implications of using FOP labels. Moreover, since the findings indicate that use of FOP labels on food products increases as level of perceived importance of healthy nutrition increases, educational nutrition programs at schools should emphasize the importance of healthy nutrition. In fact, following the 2016 reform FOP labels in Chile, mothers in that country agreed that the schools have become key promoters of food behavioral change [7].

The current study has some limitations. One of the limitations is that the telephone survey included recall questions. Recalled self-reported answers can be inaccurate and affected by the social desirability bias, especially with respect to nutrition behavior [21]. Therefore, the actual usage of FOP labels may be different than the self-reported usage in the current study. In fact, a telephone or web survey cannot replicate a real shopping environment [22]. Nevertheless, according to Subar et al. self-reported dietary data are important and can be used successfully to inform dietary guidance and public health policy [23]. Another potential limitation of the study is related to the script of the consent form asking people to participate. Participants were invited to participate in a survey about attitudes toward nutrition before they were asked to give their consent. Therefore, the respondents may be over-representative of those with positive attitudes towards nutrition, which could bias the results. Nevertheless, it is customary and important to mention the topic of the telephone survey in the consent request. In addition, the response rate of 50% is relatively high, compared to the 30% response rate typical of telephone surveys of this type [24].

Another limitation concerns the representativeness of the sample. The proportion of those with more than 12 years of education (63.1%) in the sample was greater than their proportion in the population (59.7%), yet other socio-demographic characteristics of the study sample (age, gender, religious) are quite similar to those of the target Israeli adult population. Moreover, the study does not examine the use of the FOP labels for specific food products and does not make a distinction between red and green labels in the HBM constructs. It is possible that the HBM constructs operate differently with respect to positive and negative health messages. Nevertheless, the survey reflects participants' attitudes and provides an important perspective on the extent of success of the new FOP label reform. Finally, the survey took place during the COVID-19 pandemic, which coincided with the policy rollout. The pandemic changed food consumption behavior and may have influenced attention, awareness and response to the policy. Future research should examine the impact of positive versus negative health messages on actual behavior and consumers' food product choices in supermarkets and food stores. Future research should also examine consumers’ long-term purchasing changes.

## Conclusions

The study found that 58.5% reported using the FOP labels to some extent. In addition, 70% indicated they would be willing to change to healthier products in the coming year. The results also show that the frequency of using FOP labels increases as the following factors increase: levels of perceived benefits and perceived importance of reading FOP labels, level of perceived importance of healthy nutrition, frequency of conforming to healthy nutrition, and support for the reform. The study's findings are important for understanding the impact of the new reform and for guiding future complementary actions to increase people’s motivation to use FOP labels. More advertisement about the FOP labels in the media and also through the HMOs is needed to increase people’s awareness of the reform. In addition, information about the reform provided to the public should emphasize the benefits and health implications of using FOP labels.

## Data Availability

The dataset supporting the conclusions of this article is available from the authors upon request.

## Appendix 1

### Participants' consent

Hello, my name is _________ and I am appealing to you as part of a study funded by the Ministry of Health on nutrition. Your opinion is very important to us and we would very much appreciate if you could participate in our survey. We guarantee that all your answers will be kept confidential and will be used for research purposes only. All data we collect will be completely anonymous, so it is not possible to link your personal information to your answers. Feel free to skip any question you do not want to answer.

Do you agree to participate in the study? 1. Yes 2. No.

## Appendix 2

See Table 5.

**Table 5:** HBM related to Front-of-Package red/green labels

Categories	Items
Perceived benefits (alpha = 0.70)	Front-of-Package red/green labels prevent fraud in food products
	Front-of-Package red/green labels provide useful information
	Front-of-Package red/green labels guarantee food quality and safety
Perceived barriers	Reading Front-of-Package red/green labels takes more time than consumers can spare
Perceived importance of reading FOP red/green labels	Using red/green labels to choose foods is better than relying on one’s own knowledge about what is in them
Perceived nutrition importance (alpha = 0.84)	It is important to take nutrition into account when shopping for food
	It is important to choose a diet low in saturated fat
	It is important to choose a diet low in sodium
	It is important to choose a diet low in sugar
	It is important to maintain a healthy weight
Health motivation	I get periodic examinations every year, in addition to visiting the doctor when I am ill
	I usually follow medical recommendations because I believe they will improve my health

## Abbreviations

FOP: Front-of-Package
HBM: Health Belief Model
ICDC: Israel Center for Disease Control
CBS: Central Bureau of Statistics
